# Longitudinal Analysis of Tetanus- and Influenza-Specific IgG Antibodies in Myeloma Patients

**DOI:** 10.1155/2012/134081

**Published:** 2012-03-12

**Authors:** Sebastian Kobold, Tim Luetkens, Britta Marlen Bartels, Yanran Cao, York Hildebrandt, Orhan Sezer, Henrike Reinhard, Julia Templin, Katrin Bartels, Nesrine Lajmi, Friedrich Haag, Carsten Bokemeyer, Nicolaus Kröger, Djordje Atanackovic

**Affiliations:** ^1^Department of Internal Medicine II, and Department of Oncology, Hematology, Bone Marrow Transplantation Section of Pneumology, University Medical Center Hamburg-Eppendorf, 20246 Hamburg, Germany; ^2^Hubertus Wald Tumorzentrum, University Cancer Center Hamburg, University Medical Center Hamburg-Eppendorf, 20246 Hamburg, Germany; ^3^Division of Clinical Pharmacology, Department of Internal Medicine, Ludwig-Maximilian University, 80336 Munich, Germany; ^4^Department of Stem Cell Transplantation, University Medical Center Hamburg-Eppendorf, 20246 Hamburg, Germany; ^5^Institute for Immunology, University Medical Center Hamburg-Eppendorf, 20246 Hamburg, Germany

## Abstract

*Background*. Multiple myeloma (MM) and its therapies may induce a severely compromised humoral immunity. We have performed a longitudinal analysis of IgG-antibody responses against influenza virus (FLU) and tetanus toxoid (TT) as surrogate markers for the B cell-mediated immunity in MM patients. *Methods*. 1094 serum samples of 190 MM patients and samples from 100 healthy donors were analyzed by ELISA for FLU- and TT-specific antibodies. *Results*. MM patients evidenced lower levels of FLU- and TT-specific antibodies than healthy controls (*P* < 0.001). Immunoreactivity decreased with progressing disease and worsening clinical status. Levels of FLU- and TT-specific antibodies increased shortly (0-6 months) after alloSCT (*P* < 0.001), a time-period during which intravenous immunoglobulin (IVIG) is routinely applied. Thereafter, antibody concentrations declined and remained suppressed for 3 years in the case of FLU-specific and for more than 5 years in the case of TT-specific antibodies. *Conclusions*. We found that MM is associated with a profound disease- and therapy-related immunosuppression, which is compensated for a few months after alloSCT, most likely by application of IVIG. This and the differences regarding the recovery of anti-FLU and anti-TT antibody titers during the following years need to be taken into account for optimizing IVIG application and immunization after alloSCT.

## 1. Introduction

Multiple myeloma (MM) is a disease arising from a malignant plasma cell clone proliferating in the bone marrow (BM) [1]. On the one hand, the growing tumor mass leads to a reduction of normal hematopoiesis, and, secondly, myeloma cells create a cytokine/chemokine microenvironment favoring the malignant phenotype while suppressing local and systemic immunity [2]. Both factors contribute to the profound immune dysregulation present in myeloma patients [3]. MM patients evidence phenotypic and functional defects of humoral as well as cellular immunity. Particularly B-cell responses are altered to a state of functional hypogammaglobulinemia, leading to an increased risk for opportunistic infections in MM patients [3]. As a consequence of the impaired protective immunity against bacteria and viruses, infections represent a major cause of death in MM patients [4]. The administration of intravenous immunoglobulin (IVIG) has been used to temporarily restore antibody-mediated immunity, in particular after high-dose chemotherapy [5]. However, it is unknown to what extent and for how long the passively transferred humoral immunity compensates the severe disease- and therapy-related immunosuppression in myeloma patients.

As MM itself is dependent on the suppression and dysregulation of the adaptive immune response, the development of different modes of immunotherapy seems an attractive option for improving treatment of myeloma patients. Allogeneic stem cell transplantation is one of the most promising ways to restore the ability of the immune system to recognize and destroy MM cells [6]. The transfer of a healthy, donor-derived immune system, which is not tolerant to the malignant plasma cell clone, is currently the only potentially curative approach for MM patients [6]. The immunological graft-versus-myeloma effect (GvM) is powerful, but it comes along with a significant risk of developing a graft-versus-host reaction (GvHD), which represents a potentially deadly threat often requiring strong prophylactic (and sometimes therapeutic) immunosuppression [6, 7]. Optimized strategies are needed to determine exactly how much immunosuppression is needed to dampen harmful alloimmune reactions while still allowing for clinically required graft-versus-myeloma effects [7].

In order to improve our understanding of the therapy- and disease-related defects in the humoral immunity of myeloma patients, antibody responses need to be assessed repeatedly during the course of the disease. Memory immune responses induced by routine vaccinations or natural exposure, like the ones directed against influenza virus (FLU) and tetanus toxoid (TT), can serve as markers for the general immune competence of the patient at a given time point [8, 9]. Surprisingly, data on the longitudinal behavior of immune responses to such common antigens are scarce and have not been systematically addressed in MM patients [10, 11], in particular not after alloSCT. Here, we have performed the largest longitudinal analysis of FLU- and TT-specific antibody responses in MM patients to evaluate the consequences of the malignancy itself as well as of different modes of therapy on humoral immunity.

## 2. Material and Methods

### 2.1. Patients

Patients were admitted for diagnostic purposes and/or treatment to the University Medical Center Hamburg-Eppendorf. Repeated blood samples were obtained during routine diagnostic procedures, and all participants provided informed consent prior to sample collection. A total of 1094 peripheral blood (PB) plasma samples were collected from 194 consecutive MM patients. In addition, 100 PB sera were obtained from healthy donors. Samples were collected as previously described [12, 13]. Patients were included between December 2004 and February 2008. All patients were diagnosed and treated for MM. None of the patients had received new agents such as bortezomib, lenalidomide, or thalidomide. AutoSCT was generally performed twice in a tandem setting. Induction treatment for alloSCT routinely comprised 140 mg/m^2^ melphalan, 150 mg/m^2^ fludarabine, and 30–60 mg anti-thymocyte globulin per kg. Peripheral hematopoietic grafts were used for transplantation and cyclosporine A (until day 180) and mycophenolate mofetil (until day 54) were used as GVHD prophylaxis. No patient received consolidation or maintenance treatment. Only single patients received sporadically received donor lymphocyte infusions. All patients received i.v. immunoglobulins on day 1, 7, 14, 21, 28, 54, and 86 after alloSCT. Booster vaccines were applied one year after alloSCT for FLU and TT, respectively. 

This study was conducted in accordance with the declaration of Helsinki. The protocol had received approval by the local ethics committee (decision number OB-038/06).

### 2.2. Proteins and Peptides

Recombinant influenza nucleoprotein (FLU) produced in *E. coli* was obtained from Imgenex (San Diego, CA, USA) and tetanus toxoid (TT) was provided by Chiron Behring (Marburg, Germany). Control protein for FLU and TT antibody detection was GST expressed in *E. coli* (Cell Systems, St. Katharinen, Germany).

### 2.3. Enzyme-Linked Immunosorbent Assay (ELISA)

ELISA was performed as previously described [13]. For all samples, the GST background value was subtracted from the FLU- or TT-specific OD at 405 nm.

### 2.4. Statistical Analysis

Statistical analyses were performed using GraphPad software. To avoid bias by repeated sampling, samples were stratified according to the time frame during which they were sampled (e.g., before alloSCT or 3 months after alloSCT) and a mean value was calculated for all samples of a given patient collected within a given time frame. In a second step, these values were used to determine the mean for the respective group of patients (i.e., all myeloma patients per time frame) as suggested by Bland and Altman [14, 15]. The Mann-Whitney U test was used to calculate differences between different patient cohorts. Analysis of covariance was used to assess correlations between FLU- and TT-specific antibodies. Correlations between clinicopathological variables and FLU- or TT-specific antibodies were determined by Pearson's *χ* ^2^  test. All tests were performed as univariate analyses. Differences were regarded significant if *P* < 0.05.

## 3. Results

### 3.1. Myeloma Patients Evidence Reduced Levels of FLU- and TT-Specific IgG Antibodies Compared to Healthy Controls

Over a time course of 4 years, a total of 194 consecutive MM patients were included into this study, and from the respective patients, 1094 PB samples were collected. A mean number of 5.4 (range 1–47) serum samples were collected per patient during a median follow-up period of 11.4 months (range 1–39 months). Most patients were included at advanced stages of the disease (mainly stage II and III according to the Salmon and Durie classification), and all but 10 patients had received chemotherapy, autologous stem cell transplantation (autoSCT), or alloSCT, respectively, as maximum therapy prior to study inclusion (see Table 1 for patient characteristics).

**Figure 1:**
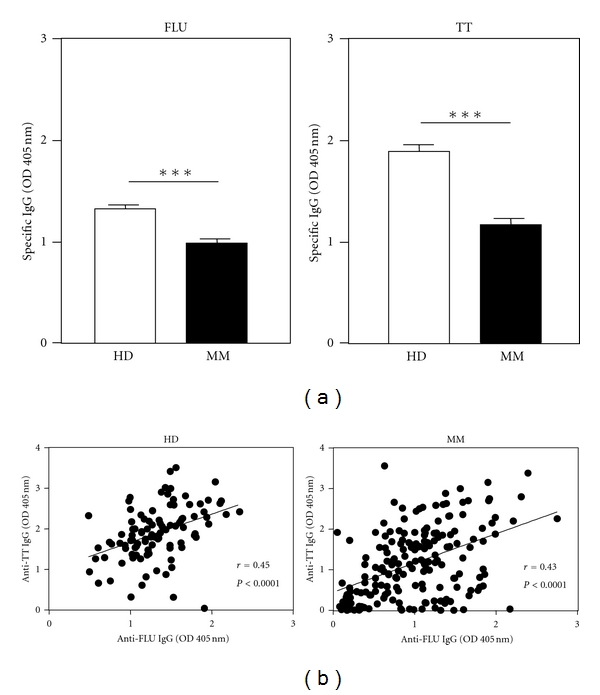
Comparison of levels of FLU- and TT-specific antibodies in MM patients compared to healthy donors. (a) Mean values for FLU- and TT-specific specific antibodies for HD (*n* = 100) and MM patients (*N* = 190). OD 405 nm of the background control GST was subtracted for each sample. Asterisks indicate significant differences (***P < 0.001) between groups. (b) Correlational analysis of FLU- and TT-specific antibodies in HD (*N* = 100) and MM patients (*N* = 190).

When we compared levels of IgG antibodies directed against FLU or TT between myeloma patients and healthy donors (*N* = 100), we found both types of humoral responses to be significantly reduced in the patients (Figure 1(a)). To address if the FLU- and TT-specific antibodies reflected the general humoral capacity of the given group of subjects to a comparable extent, we performed correlational analyses. Indeed, we observed that levels of FLU- and TT-specific IgG antibodies correlated positively and highly significantly in patients as well as those in the group of donors (Figure 1(b)). This finding further indicated that a state of general immunosuppression was present in the patients, irrespective of the nature of the given antigen. It is important to note, however, that myeloma patients were compared to unselected, anonymized blood donors and that we, therefore, cannot rule out that differences observed were partly related to confounding factors, that is, the median age of each group of subjects.

### 3.2. FLU and TT Specific Antibodies Show a Transient Increase Followed by a Long-Lasting Suppression after AlloSCT

**Figure 2:**
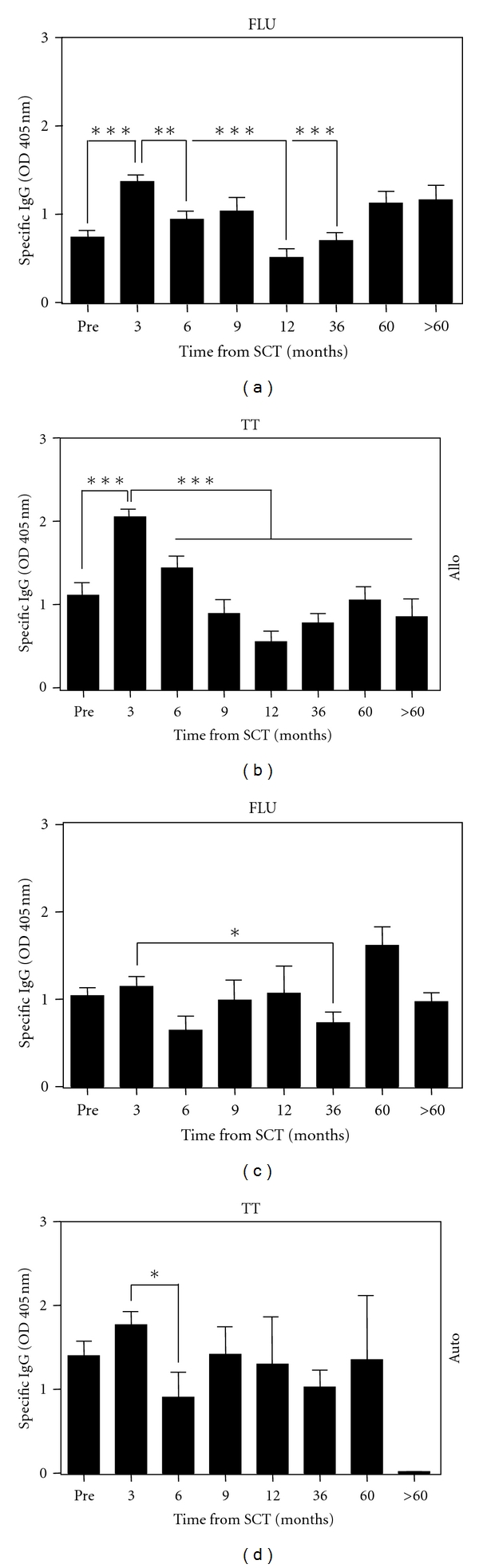
Time course of FLU- and TT-specific antibodies in MM patients undergoing alloSCT. Samples harvested in the frame of allo- and autoSCT were sorted according to time after transplantation. Only those patients were included into this analysis who had alloSCT or autoSCT, as maximum therapy. For the samples collected in the frame of alloSCT the group numbers of samples per time point were as follows: 30, 24, 24, 18, 19, 45, 33, and 20. For the samples collected in the frame of alloSCT, the group numbers of samples per time point were as follows: 25, 21, 10, 10, 4, 18, 2, and 2. Asterisks indicate significant differences when compared to the time point “3 months” after SCT (**P < 0.01 and ***P < 0.001).

Since both alloSCT and autoSCT are known to have significantly impact on the immune capacity of the patient, we asked how IgG antibody responses against FLU and TT are influenced by each type of transplantation. Only such patients were included in this analysis who had either received autoSCT or alloSCT as maximum therapy. When we monitored levels of FLU- and TT-specific antibodies before and after autoSCT, we did not find any major changes during the follow-up period when compared to pretransplant values (Figure 2). In contrast, both FLU and TT antibodies significantly increased during the first three months after alloSCT to a level comparable to healthy donors. Thereafter, humoral responses declined significantly and remained suppressed for 3 and more than 5 years in the case of FLU- and TT-specific antibodies, respectively.

**Figure 3:**
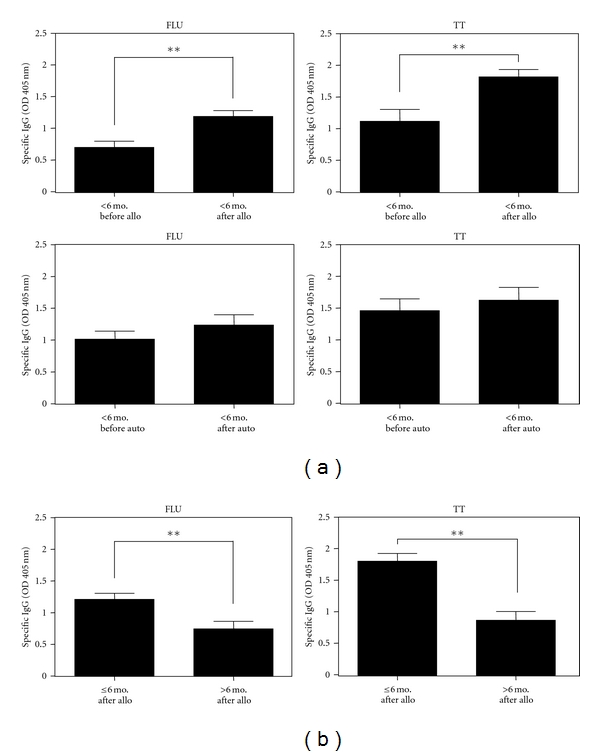
Time-dependent impact of alloSCT on FLU- and TT-specific antibodies in selected patients. (a) Comparison of FLU and TT antibodies 6 months before and 6 months after alloSCT and autoSCT. For 21 alloSCT patients and for 14 autoSCT patients, samples had been collected for both of these time points. Differences were significant with *P* < 0.001 for FLU-antibodies before and after alloSCT and with *P* < 0.0001 for TT antibodies before and after alloSCT. No significant differences were found between FLU and TT antibody concentrations before and after autoSCT. (b) Comparison of FLU and TT antibodies collected less than 6 months after alloSCT and more than 6 months after alloSCT. Samples had been collected from 20 alloSCT patients at both time points. Differences in antibody titers were significant in the case of anti-FLU (*P* < 0.01) and anti-TT antibodies (*P* < 0.0001).

To analyze the influence of alloSCT on humoral immunity against FLU and TT in a more detailed manner, we selected a cohort of patients of whom we had been able to obtain samples during the last 6 months before alloSCT and the first 6 months after alloSCT. Comparing levels of FLU- and TT-specific antibodies for this homogenous collective at both time points, we confirmed our observation of a significant increase following alloSCT (Figure 3(a)). In contrast, no significant change was found when we compared the same time points in a group of patients who had received autoSCT (Figure 3(a)). Next we compared samples of the same patients, collected at time points equal 6 months or less after alloSCT with samples collected more than 6 months after alloSCT. We were able to demonstrate that 6 months represent an important cutoff with regard to the humoral immunity of MM patients after alloSCT (Figure 3(b)). In contrast no difference in FLU- or TT-specific antibodies was found when autoSCT patients were monitored over the same period of time (data not shown).

### 3.3. Levels of FLU and TT Antibodies Correlate Negatively with Markers of Poor Prognosis in MM

As a next step, we correlated FLU and TT antibody levels with a large variety of clinicopathological measures (Table 2). For FLU-specific antibodies, the only statistically significant association was found for concentrations of total IgG in IgG myeloma with lower IgG concentration being associated with elevated anti-FLU antibody levels (Table 2). On the other hand, higher levels of TT-specific antibodies were significantly associated with younger age (<60 years) and normal serum calcium and albumin as well as normal IgG concentrations in IgG myeloma. Overall, these associations suggest that general immunoreactivity decreases with progressing disease and worsening clinical status of the patient.

## 4. Discussion

Analyzing the largest cohort of MM patients to date for the presence of FLU- and TT-specific antibodies, we found myeloma patients to evidence significantly reduced levels of antibodies against both antigens. MM patients are known to be deficient in polyclonal immunoglobulins [16]. Upon vaccination MM patients show a delayed increase in IgM, a quicker shift to IgG and lower titers of antibodies against the target antigen [17]. The general B-cell dysfunction in myeloma patients results in antibody titers below protective levels, rendering MM patients more susceptible to infections despite vaccination [3, 18]. Levels of FLU- and TT-specific antibodies have been investigated in small cohorts of MM patients, mostly revealing lower antibody titers in MM than in healthy controls [18–21]. In our large longitudinal study, we were able to confirm these results and we believe that these reduced levels of antibodies against two selected targets indeed reflect the negative impact of the malignancy and previous therapies on the humoral (and probably also cellular) immunity in myeloma patients. This idea would also be supported by our observation of a negative correlation between TT- and/or FLU-specific antibody levels and markers of a poor prognosis in MM such as paraprotein levels and concentrations of serum calcium and albumin as well as the patient's age.

In addition to the aforementioned parameters we detected a strong influence of alloSCT on the levels of TT- and FLU-specific IgG antibodies in myeloma patients. We observed elevated antibody levels during the first 6 months after alloSCT followed by a suppression of humoral immunity for up to 5 years and more. Treatments such as chemotherapy, autoSCT, and alloSCT have previously been shown to significantly influence the immune system of cancer patients [9, 10, 22, 23]. In MM, small studies have suggested alloSCT to impair the humoral immune response, but little is known about the time course of this suppression, and data on ideal time points for vaccination are controversial [11, 24]. In our current study, we were able to demonstrate that, as in other diseases requiring alloSCT, immunity to FLU and TT decreases over time following transplantation, most likely as a surrogate marker of prolonged immunosuppression [25]. On the other hand, we also found a transient increase of FLU and TT antibodies in the first 6 months following alloSCT. While there are a number of possible explanations for this increase, we believe that it is most likely caused by intravenous substitution with polyclonal intravenous immunoglobulins (IVIGs) commonly performed at our center during the first months post transplantation [26]. This hypothesis would be consistent with previous studies describing large amounts of both FLU- and TT-specific antibodies as parts of IVIG preparations [27].

Another interesting observation we have made in our current analysis is that, following the initial amplification of humoral immune responses against TT and NP, antibody levels were suppressed for at least 3 years in the case of FLU-specific immunity while TT-specific antibodies remained below early post-alloSCT (<6 months) levels for the whole remaining observation period. There are four possible explanations for the discrepancies in the behavior of both antibody specificities. First, based on the fact that FLU-specific immunity is often acquired spontaneously while TT-specific humoral responses are always generated by vaccination, that repeated natural exposures to influenza antigen may have boosted the antibody response [8, 18]. On the other hand, TT-specific vaccination may have less stringently been performed than FLU-specific vaccination because, in contrast to influenza infections, tetanus infections are not a leading cause of mortality in MM patients. This hypothesis is least likely to fit, since at our institutions, TT antibodies are routinely monitored and low titers lead to repeated booster vaccinations. Third, Ek et al. have previously reported that in leukemia patients levels of TT- but not *Haemophilus influenzae*-specific antibodies correlated negatively with disease recurrence and were less protective in these immunosuppressed patients [8] indicating that the extent of treatment-induced humoral immunosuppression might indeed depend on the type of the given antigen [8]. Accordingly, it has previously been described that antiviral antibody responses were more stable with a half-life of up to 50 years compared to 10 years for antibacterial responses, that is, against TT [28]. We, therefore, believe that the latter concept is most likely to explain the different time-frames until recovery of TT- and NP-specific antibodies after alloSCT.

Overall, our current findings support the concept that MM is associated with a profound disease- and therapy-related immunosuppression which is compensated for a few months after alloSCT by the application of intravenous immunoglobulin. This and the fundamental differences regarding the recovery of anti-FLU and anti-TT antibody titers during the following years need to be taken into account for optimizing strategies for IVIG application and active immunization after alloSCT.

## Figures and Tables

**Table 1:** Patient characteristics. Data are shown for all patients. LC: light chain, HC: heavy chain. ^§^indicates missing information for some patients.

Parameter	Total
Sex	
Male	115
Female	75

Age	

> 60	69
≤ 60	121

Karyotype	

Normal	83
Complex	15
del13q14	46
del17p13	12
*t *(4; 14)	9
Not tested	25

LC isotype	

Light lambda	62
Light kappa	100

HC isotype^§^	

IgG	167
IgA	18

Maximum treatment	

Untreated	10
Chemotherapy	81
autoSCT	30
alloSCT	74

Stage^∗,#,§^	

I	32
II	52
III	95

*One patient was found to bear a 13q14 and a 17p13 deletion.

^#^Stage according to the Salmon and Durie classification.

**Table 2:** correlation of FLU-antibodies and TT-antibodies with clinical parameters.

Parameter	Stratification	*N*	Mean (FLU)	Mean (TT)
Gender	men	115	1,042	1,202
	women	75	0,918	1,133

Age	≤ 60 years	122	0,969	1,253*
	> 60 years	67	1,034	1,023*

Hemoglobin	low	157	1,022	1,181
	normal	30	0,859	1,087

Albumin	<35 g/l	27	0,877	0,699*
	≥35 g/l	158	1,015	1,247*

LDH	≤225 U/I	137	1,007	1,156
	>225 U/I	49	0,972	1,194

Calcium	≤2,63 mmol/l	182	0,995	1,145*
	>2,63 mmol/l	2	1,040	2,470*

Creatinin	≤1,3 mg/dL	146	0,990	1,123
	>1,3 mg/dL	37	1,035	1,355

IgG (for IgG myeloma)	≤16 g/l	44	1,018	1,248*
	>16 g/l	41	0,746	0,874*

IgG (for IgA myeloma)	≤16 g/l	39	0,994	1,103
	>16 g/l	4	0,883	0,870

IgA (for IgA myeloma)	≤4 g/l	17	0,986	1,151
	>4 g/l	26	0,982	1,034

Kappa-light chains	≤3,7 g/l	14	1,116	1,557
	>3,7 g/l	0	—	—

Lambda-light chain	≤2 g/l	12	1,164	1,007
	>2 g/l	1	0,210	0,030

Deletion 13q14	positive	46	0,900	1,014
	negative	117	1,038	1,218

Deletion 17p13	positive	13	1,058	1,350
	negative	150	0,989	1,142

Translocation *t *(4; 14)	positive	9	0,933	0,793
	negative	153	0,998	1,178

**β**2-Microglobulin	≤3 mg/l	60	0,970	1,200
	>3 mg/l	32	0,807	0,942

GvHD	positive	42	0,885	0,965
	negative	21	0,893	1,172

Plasma cells in BM	≤10%	68	0,978	1,241
	>10%	37	0,949	1,006

*indicates a statistically significant result (*P* < 0.05); if not otherwise specified, differences between groups are not significant.
